# Ethanolic Extract of Polish Propolis: Chemical Composition and TRAIL-R2 Death Receptor Targeting Apoptotic Activity against Prostate Cancer Cells

**DOI:** 10.1155/2013/757628

**Published:** 2013-11-12

**Authors:** Ewelina Szliszka, Anna Sokół-Łętowska, Alicja Z. Kucharska, Dagmara Jaworska, Zenon P. Czuba, Wojciech Król

**Affiliations:** ^1^Department of Microbiology and Immunology, Medical University of Silesia in Katowice, Jordana 19, 41 808 Zabrze, Poland; ^2^Department of Fruit and Vegetables and Cereals Technology, Wrocław University of Environmental and Life Sciences, Chełmońskiego 47/41, 51 630 Wrocław, Poland

## Abstract

Propolis possesses chemopreventive properties through direct anticancer and indirect immunomodulatory activities. Tumor necrosis factor-related apoptosis-inducing ligand (TRAIL) plays a significant role in immunosurveillance and defense against cancer cells. TRAIL triggers apoptosis upon binding to TRAIL-R1 (DR4) and TRAIL-R2 (DR5) death receptors expressed on cancer cell surface. The activation of TRAIL apoptotic signaling is considered an attractive option for cancer prevention. However, as more tumor cells are reported to be resistant to TRAIL-mediated death, it is important to develop new strategies to overcome this resistance. The aim of this study was to investigate the chemical composition and proapoptotic mechanism of ethanolic extract of Polish propolis (EEP-P) against cancer cells. The identification and quantification of phenolic compounds in propolis extract were performed using HPLC-DAD and UPLC-Q-TOF-MS methods. TRAIL-resistant LNCaP prostate cancer cells were treated with EEP-P and TRAIL. Cytotoxicity was measured by MTT and LDH assays. Apoptosis was detected using annexin V-FITC staining by flow cytometry and fluorescence microscopy. Death receptors expression was analyzed using flow cytometry. Pinobanksin, chrysin, methoxyflavanone, *p*-coumaric acid, ferulic acid and caffeic acid were the main phenolics found in EEP-P. Propolis sensitized LNCaP cells through upregulation of TRAIL-R2. These results suggest that EEP-P supports TRAIL-mediated immunochemoprevention in prostate cancer cells.

## 1. Introduction

The field of cancer prevention, defined as the long-term intervention with natural or synthetic molecules to inhibit, delay, or reverse carcinogenesis, is gaining increasing importance, especially at the time when use of complementary and alternative medicine (CAM) and natural health products is consistently growing. At present, CAM in oncology represents a challenging area of interest since remarkable scientific evidence suggests that natural agents can prevent the process of carcinogenesis and effectively influence the risk of cancer in humans [1–3]. Prostate cancer represents an ideal disease for chemopreventive strategies because of its long latency, late age of onset, relatively slow rate of growth and progression, high incidence, tumor marker availability, and identifiable preneoplastic lesions and risk groups [4, 5].

Propolis (bee glue) is a resinous hive product collected by honeybees from many plant sources. The chemical composition of propolis is complex and largely depends on the geographical origin and specific flora at the site of collection [6, 7]. The main phenolics found in Polish propolis are phenolic acids, including cinnamic, *p*-coumaric, ferulic, and caffeic acid, caffeic acid phenylethyl ester (CAPE), and flavonoids, such as chrysin, tectochrysin, apigenin, pinocembrin, pinostrombin, pinobanksin, galangin, kaempferol, kaempferide, and quercetin [8–11]. Propolis cannot be used as raw material, and it must be purified by extraction to remove the inert material and preserve the phenolic fraction [9]. The ethanolic extract of propolis (EEP) exhibits chemopreventive properties through direct anticancer and indirect immunomodulatory properties [9, 12, 13]. EEP suppresses proliferation and tumor growth and induces cell-cycle arrest and apoptosis in prostate cancer cells [9, 14].

Tumor necrosis factor-related apoptosis-inducing ligand (TRAIL), a member of the TNF superfamily, is a powerful inducer of apoptosis in cancer cells without toxicity against normal tissues [15, 16]. The death ligand is expressed on T lymphocytes, natural killer cells, neutrophils, monocytes, or macrophages [17, 18]. Membrane-bound TRAIL can be cleaved from the cell surface into a soluble secreted form. Soluble or expressed on immune cells TRAIL plays an important role in surveillance and defense against tumor cells [19]. Endogenous TRAIL triggers apoptosis *via *receptor-mediated death through interaction with the death receptors (DRs) in cancer cells [20, 21]. TRAIL initiates programmed death upon binding to TRAIL-R1 (DR4) and TRAIL-R2 (DR5) receptors and promotes recruitment of the adaptor molecule Fas-associated death domain (FADD) with formation of the death inducing signaling complex (DISC) and subsequent activation of the caspases cascade [22, 23].

The induction of cancer cell-specific apoptosis *via* the activation of TRAIL signaling has become an important focus of cancer research [24, 25]. However, some cancer cells are resistant to TRAIL-induced death. Failure to undergo apoptosis has been implicated in resistance of cancer cells to TRAIL surveillance, tumor development, and progression. Multiple factors contribute to TRAIL resistance, including disorder in expression of DRs and proapoptotic or antiapoptotic proteins [26, 27]. As more tumor cells are reported to be resistant to TRAIL-mediated death, it is needed to develop new strategies to overcome this resistance [28, 29]. Polish and Brazilian EEP have been shown to sensitize prostate cancer cells to TRAIL-induced apoptosis [9, 30]. TRAIL-R2 called death receptor 5 (DR5) or “KILLER” receptor is a crucial player in the transduction of apoptotic signaling in cancer cells derived from solid tumors [31, 32]. We hypothesize that this immunomodulation through targeting of TRAIL-R2 death receptor by propolis extracts is one of the mechanisms responsible for its cancer preventive effect.

The major aim of this study was to determine the chemical composition and the proapoptotic mechanism of ethanolic extract of Polish propolis (EEP-P) against cancer cells. We investigated the involvement of TRAIL-R2 in EEP-P modulation of TRAIL-mediated apoptotic signaling in LNCaP prostate cancer cells.

## 2. Materials and Methods

### 2.1. General

Soluble recombinant human TRAIL was purchased from PeproTech Inc. (Rocky Hill, NJ, USA). Acetonitrile, formic acid, and dimethyl sulfoxide (DMSO) were obtained from Sigma-Aldrich (Steinheim, Germany). Acetonitrile for LC-MS was purchased from POCh (Gliwice, Poland). The following compounds were used as standards: caffeic acid and rhamnetin (Roth, Karlsruhe, Germany), apigenin, chrysin, galangin, pinobanksin, and *p*-coumaric acid (Sigma-Aldrich, Steinheim, Germany), and ferulic acid (Serva, Heidelberg, Germany).

### 2.2. Preparation of Polish Propolis Extract

Propolis was collected manually from beehive located in southern region in Poland (The Carpathians) and was kept desiccated pending its processing. As previously described, propolis sample was extracted in 95% v/v ethanol at 37°C for 4 days, in a hermetically closed glass vessel under occasional shaking. The ethanolic extract of Polish propolis (EEP-P) was then filtered through a Whatman filter paper no. 4 and evaporated in a rotary evaporator under reduced pressure at 60°C. The same collection and extraction procedures were used throughout all our laboratory studies [9]. EEP-P was dissolved in DMSO (50 mg/mL), and the final concentration of DMSO in the culture medium was controlled at 0.1% (v/v). 

### 2.3. Identification and Quantification of Phenolic Compounds by HPLC-DAD Method

 Phenolic compounds were determined using Dionex (USA) HPLC system equipped with diode array detector model Ultimate 3000, a quaternary pump LPG-3400A, autosampler EWPS-3000SI, and thermostated column compartment TCC-3000SD and controlled by Chromeleon v.6.8 software. The reversed phase Cadenza 5CD-C18 (75 mm × 4.6 i.d.) column (Imtakt, Kyoto, Japan) with guard column Cadenza (5 × 4.6 i.d.) guard column (Imtakt, Kyoto, Japan) was used. The mobile phase was composed of (A) 0.1% (v/v) formic acid in water and (B) acetonitrile. The applied elution conditions were 0 min 20% B; 0–10 min linear gradient from 20% to 30% B; 10–40 min linear gradient from 30% to 40% B; 40–60 min, linear gradient from 40% to 60% B; 60–80 min, linear gradient from 60% to 80% B; and then again the initial conditions [33]. The flow rate was 1 mL/min, and the injection volume was 20 *μ*L. The column was operated at 30°C. The compounds were monitored at 290 nm, 325 nm, and 370 nm.

### 2.4. Identification and Quantification of Phenolic Compounds by LC–MS Method

Compounds identification was performed on an Acquity ultraperformance liquid chromatography (UPLC) system coupled with a quadruple-time of flight (Q-TOF) MS instrument (UPLC/Synapt Q-TOF MS, Waters Corp., Milford, MA, USA) with an electrospray ionization (ESI) source. Separation was achieved on the Acquity BEH C18 column (100 mm × 2.1 mm i.d., 1.7 *μ*m; Waters). Detection wavelengths were set at 290, 325, and 370 nm. A mobile phase was a mixture of 1.5% formic acid (A) and acetonitrile (B). The gradient program was as follows: initial conditions—95% (A), 12 min—5% (A), 13 min—5% (A), 14.5 min—95% (A), and 16 min—95% (A). The flow rate was 0.45 mL/min, and the injection volume was 5 *μ*L. The column was operated at 30°C. The major operating parameters for the Q-TOF MS were set as follows: capillary voltage 2.0 kV, cone voltage 45 V, cone gas flow of 11 L/h, collision energy 50 eV, source temperature 100°C, desolvation temperature 250°C, collision gas, argon; desolvation gas (nitrogen) flow rate, 600 L/h; data acquisition range, *m*/*z* 100–1.000 Da; ionization mode, negative [34]. The data were collected by Mass-Lynx V 4.1 software.

### 2.5. Cell Culture

The human androgen-dependent LNCaP prostate cancer cell line was obtained from the German Collection of Microorganisms and Cell Cultures (DSMZ, Braunschweig, Germany). Cells were maintained in RPMI 1640 medium supplemented with 10% heat-inactivated fetal bovine serum, 4 mM L-glutamine, 100 U/mL penicillin, and 100 *μ*g/mL streptomycin at 37°C and 5% CO_2_ in a humidified incubator [35]. Reagents for cell culture were purchased from the American Type Culture Collection (Manassas, VA, USA).

### 2.6. Cytotoxicity Assay

Cytotoxicity was measured by the 3-(4,5-dimethyl-2-thiazyl)-2,5-diphenyl-2*H*-tetrazolium bromide (MTT) assay [36]. The MTT assay is based on the cleavage of the tetrazolium salt MTT to a blue formazan dye by viable cells. LNCaP cells (2 × 10^5^/mL) were seeded 24 h before the experiments in a 96-well plate. Various concentrations of EEP-P (25–50 *μ*g/mL) with or without TRAIL (100–200 ng/mL) were added to the cells for 24–48 h. After this time, 20 *μ*L of MTT solution (5 mg/mL) was added to each well for 4 h. The resulting blue formazan crystals were dissolved in DMSO. These reagents were purchased from Sigma Chemical Company (St. Louis, MO, USA). Controls included native cells and medium alone. Spectrophotometric absorbance was measured at 550 nm wavelength using Eon Microplate Spectrophotometer (BioTek, Winooski, VT, USA). The percent cytotoxicity was calculated by the following formula: percent cytotoxicity (cell death) = (1 − [absorbance of experimental wells/absorbance of control wells]) × 100%.

### 2.7. Lactate Dehydrogenase Release Assay

 Lactate dehydrogenase (LDH) is a stable cytosolic enzyme released upon membrane damage in necrotic cells. LDH activity was detected using a cytotoxicity assay kit (Roche Diagnostics GmbH, Mannheim, Germany) [37]. LNCaP cells were treated with EEP-P (25–50 *μ*g/mL) with or without TRAIL (100–200 ng/mL) for 24–48 h. Supernatants were then removed from each sample for measurements of LDH. LDH released into the culture supernatants was detected with a coupled enzymatic assay that results in the conversion of a tetrazolium salt into a red formazan product. Spectrophotometric absorbance was measured at 490 nm wavelength using Eon Microplate Spectrophotometer (BioTek, Winooski, VT, USA). Maximal release of LDH was obtained after treating control cells with 1% Triton X-100 (Sigma Chemical Company) for 10 min at room temperature. The percentage of necrotic cells was expressed using the following formula: (sample value/maximal release) × 100%.

### 2.8. Detection of Apoptosis by Flow Cytometry

Apoptosis was determined by flow cytometry using the Apoptest-FITC Kit with annexin V (Dako, Glostrup, Denmark). LNCaP cells (2 × 10^5^/mL) were seeded in 24-well plates for 24 h prior to experimentation and then exposed to EEP-P (25–50 *μ*g/mL) and/or TRAIL (100–200 ng/mL) for 24–48 h. Then the cells were washed twice with phosphate-buffered saline solution (PBS) and resuspended in 500 *μ*L of binding buffer. The cell suspension (290 *μ*L) was incubated with 5 *μ*L of annexin V-FITC and 5 *μ*L of propidium iodide for 10 min at room temperature in the dark. The population of annexin V-positive cells was evaluated by flow cytometry (LSR II, Becton Dickinson Immunocytometry Systems, San Jose, CA, USA) [38].

### 2.9. Detection of Apoptosis by Fluorescence Microscopy

Apoptotic cells were quantified using the fluorescence microscopy method of the Apoptotic, and Necrotic, and Healthy Cells Quantification Kit from Biotium, Inc. (Hayward, CA, USA). LNCaP cells (2 × 10^5^/mL) were seeded in a 24-well plate for 24 h before the experiments. EEP-P (25–50 *μ*g/mL) and/or TRAIL (100 ng/mL) were added to the cells for 24–48 h. After this time the cells were washed with PBS, and trypsinised. Next, the cells were centrifuged to discard the supernatant, washed with PBS and resuspended in binding buffer (100 *μ*L/sample). A combination of 5 *μ*L of annexin V-FITC, 5 *μ*L of ethidium homodimer III, and 5 *μ*L of Hoechst 33342 solution was added to each tube. The samples were incubated at room temperature for 15 min in the dark, and then the cells were washed with binding buffer and placed on a glass slide. The stained cells were observed with an IX51 fluorescence inverted microscope (Olympus, Tokyo, Japan) using filter sets for FITC, TRITC, and DAPI [39]. The cells were counted, and the number of apoptotic cells was expressed as a percentage of the total number of cells.

### 2.10. Flow Cytometric Analysis of Death Receptor Expression

The cell surface expression of death receptors TRAIL-R1 (DR4) and TRAIL-R2 (DR5) was determined by flow cytometry (LSR II, Becton Dickinson Immunocytometry Systems, San Jose, CA, USA) [40]. LNCaP cells (2 × 10^5^/mL) were seeded in 24-well plates for 24 h and exposed to EEP-P (25–50 *μ*g/mL) for 12–24 h. The cells were then harvested using solution of trypsin and ethylenediaminetetraacetic acid (EDTA), washed twice in PBS, and resuspended in PBS containing 0.5% bovine serum albumin (BSA). LNCaP cells were incubated with 10 *μ*L phycoerythrin-conjugated anti-TRAIL-R1 or anti-TRAIL-R2 monoclonal antibody (R&D Systems, Minneapolis, MN, USA) at 4°C for 45 min. After staining, the cells were washed with PBS and analyzed using flow cytometry. The control sample consisted of cells in a separate tube treated with phycoerythrin-labelled mouse IgG_1_ or mouse IgG_2B_ (R&D Systems). To show that the induction of apoptosis caused by the cotreatment of EEP and TRAIL is mediated through TRAIL-R2, the TRAIL-R2/Fc chimera protein (R&D Systems) was used. TRAIL-R2/Fc acts as a dominant negative against endogenous TRAIL-R2/DR5 receptor.

### 2.11. The Statistical Analysis

The results are expressed as the mean ± SD obtained from three independent experiments performed in quadruplicate (*n* = 12) or duplicate (*n* = 6). Statistical significance was evaluated using Student's *t*-test. *P* values <0.05 were considered significant.

## 3. Results

### 3.1. The Content and Characterization of Phenolic Compounds Identified in Extract of Polish Propolis

**Figure 1:**
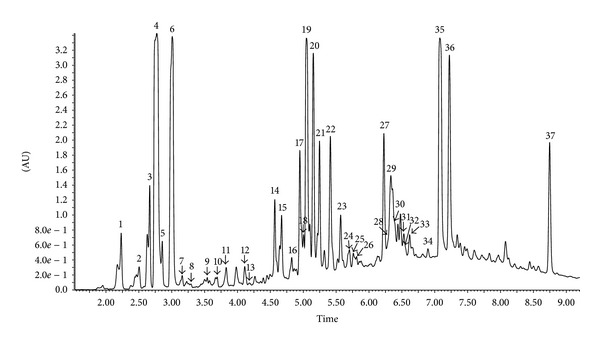
UPLC-DAD chromatogram (290 nm) of compounds of ethanol extract from Polish propolis.

**Figure 2:**
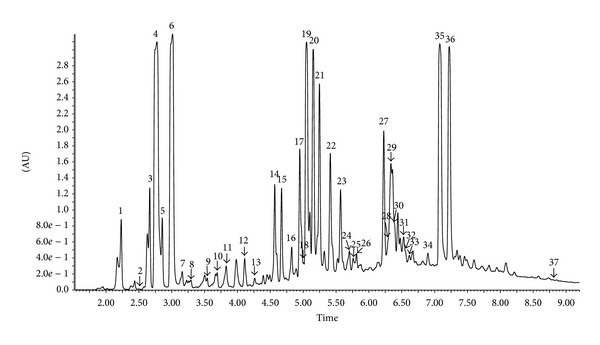
UPLC-DAD chromatogram (325 nm) of compounds of ethanol extract from Polish propolis.

**Figure 3:**
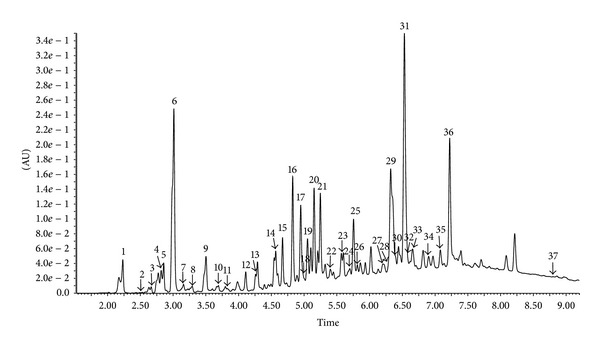
UPLC-DAD chromatogram (370 nm) of compounds of ethanol extract from Polish propolis.

**Figure 4:**
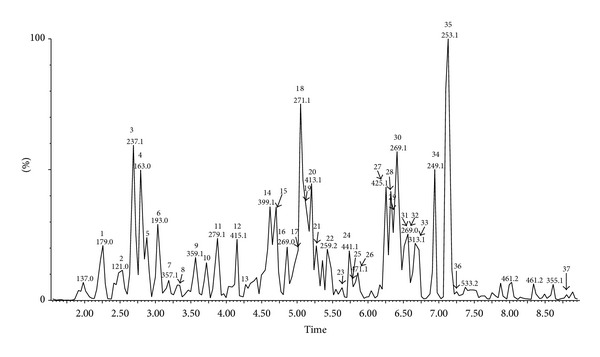
UPLC-ESI-MS (negative ion) chromatogram of main compounds of ethanol extract from Polish propolis.

The chemical composition of extract of Polish propolis was determined using HPLC-DAD and UPLC-Q-TOF-MS methods. Qualitative analysis results obtained by LC-ESI/MS methods and quantitative analysis data evaluated by HPLC (quantified using DAD detection) are presented in Figures 1, 2, 3, and 4 and Table 1. A total of thirty-seven phenolic ingredients were found in tested propolis sample. Thirty-one compounds were identified by comparison of their UV and MS/MS spectra to standards and/or to the literature data, whereas the other six compounds remained unknown. Pinobanksin, chrysin, and methoxyflavanone, which were characterized by MS from their molecular ions at *m*/*z* 271.0616, 253.0502, and 253.0806, respectively, are the major flavonoids identified in Polish propolis. Among the phenolic acids, prevailed *p-*coumaric acid (*m*/*z* 163.0406 and fragment at *m*/*z* 119 resulting from the loss of a COO group), ferulic acid (*m*/*z* 193.0492 and fragments 149.0613 and 134,0375), caffeic acid (*m*/*z* 179.0349 and fragments 161.0241 and 135.0440) and their derivatives (Table 1). 

### 3.2. Anticancer Activity of EEP-P against LNCaP Cells

**Figure 5:**
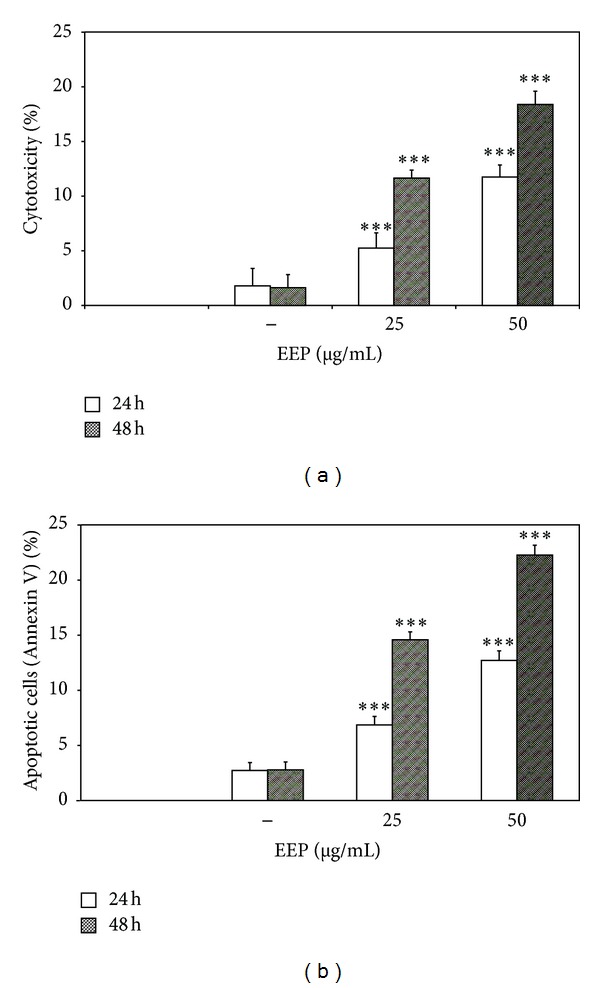
Cytotoxic and apoptotic effects of EEP-P on LNCaP prostate cancer cells. Cells were incubated with 25–50 *μ*g/mL EEP-P for 24–48 h. The values represent mean ± SD of three independent experiments performed in quadruplicate (*n* = 12). (a) Cytotoxic activity of EEP-P against LNCaP cells. The percentage of cell death was measured using the MTT cytotoxicity assay (*** = *P* < 0.001 compared to control). (b) Apoptotic activity of EEP-P against LNCaP cells. Apoptotic cell death was detected by flow cytometry using annexin V-FITC staining (*** = *P* < 0.001 compared to control).

EEP-P induced cytotoxicity and apoptosis in a dose- and time-dependent manner in LNCaP cells (Figure 5). The cytotoxic effect of 25–50 *μ*g/mL EEP-P after a 24-hour incubation was 5.2 ± 1.4% to 11.7 ± 1.1% cell death and after a 48-hour incubation was 11.7 ± 0.7% to 18.4 ± 1.2% cell death. At the same concentrations EEP-P induced 6.9 ± 0.8%–12.7 ± 0.9% (24-hour incubation) and 14.6 ± 0.7%–22.3 ± 0.9% (48-hour incubation) apoptosis in LNCaP cells. The necrotic cell death percentage of LNCaP cells incubated with 25–50 *μ*g/mL EEP-P for 24–48 h examined by Apoptest-FITC and LDH assay was near zero. 

### 3.3. EEP-P Sensitizes LNCaP Cells to TRAIL-Induced Cytotoxicity and Apoptosis

**Figure 6:**
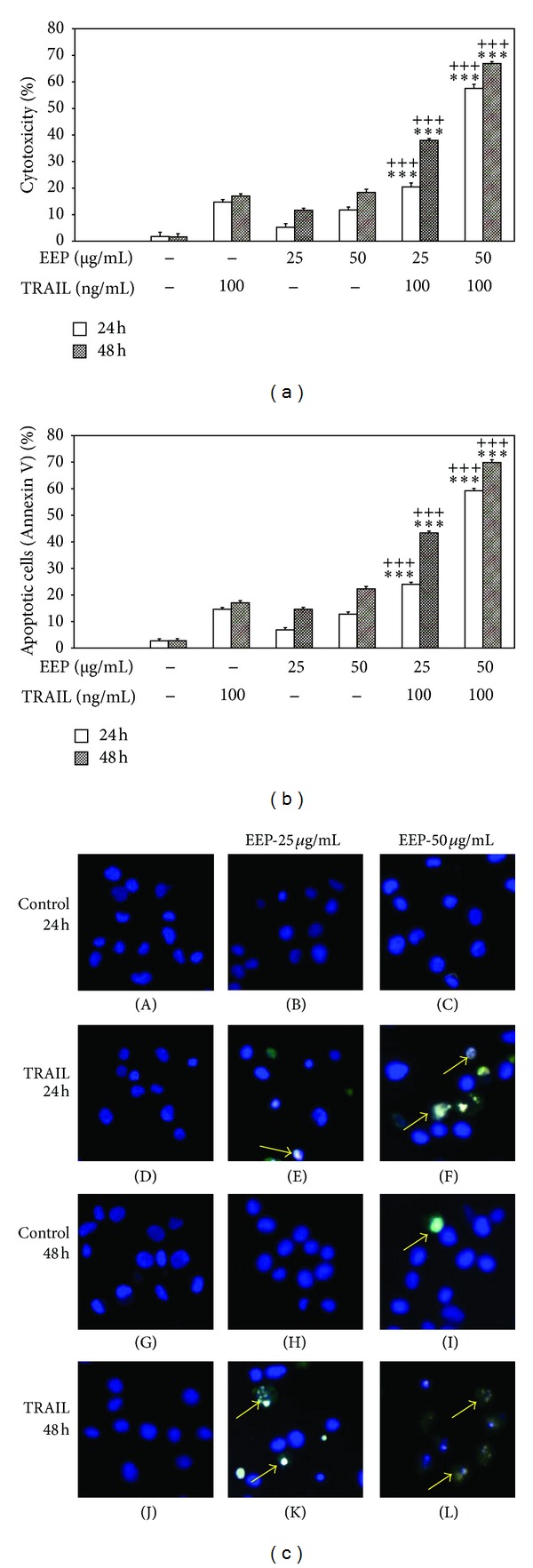
EEP-P sensitizes LNCaP prostate cancer cells to TRAIL-induced cytotoxicity and apoptosis. Cells were incubated with 25–50 *μ*g/mL EEP-P and/or 100 ng/mL TRAIL for 24–48 h. The values represent mean ± SD of three independent experiments performed in quadruplicate (*n* = 12). (a) Cytotoxic activity of EEP-P in combination with TRAIL against LNCaP cells. The percentage of cell death was measured using the MTT cytotoxicity assay (*** = *P* < 0.001 compared to EEP-P alone,  ^+++^ = *P* < 0.001 compared to TRAIL alone). (b) Apoptotic activity of EEP-P in combination with TRAIL against LNCaP cells. Apoptotic cell death was detected by flow cytometry using annexin V-FITC staining (*** = *P* < 0.001 compared to EEP-P alone,  ^+++^ = *P* < 0.001 compared to TRAIL alone). (c) Apoptotic activity of EEP-P in combination with TRAIL against LNCaP cells: (A) control cells, (B) cells incubated with 25 *μ*g/mL EEP, (C) cells incubated with 50 *μ*g/mL EEP, (D) cells incubated with 100 ng/mL TRAIL, (E) cells incubated with 25 *μ*g/mL EEP and 100 ng/mL TRAIL, (F) cells incubated with 50 *μ*g/mL EEP and 100 ng/mL TRAIL for 24 h, (G) control cells, (H) cells incubated with 25 *μ*g/mL EEP, (I) cells incubated with 50 *μ*g/mL EEP, (J) cells incubated with 100 ng/mL TRAIL, (K) cells incubated with 25 *μ*g/mL EEP and 100 ng/mL TRAIL, and (L) cells incubated with 50 *μ*g/mL EEP and 100 ng/mL TRAIL for 48 h. Apoptotic cell death was detected and visualized by fluorescence microscopy using annexin V-FITC staining. Healthy cells (stained with Hoechst 33342) emitted blue fluorescence and apoptotic cells (stained with Hoechst 33342 and annexin V-FITC) emitted green and blue fluorescence (indicated by arrows).

 The cytotoxic effect of 100 ng/mL TRAIL after a 24-hour incubation was 14.7 ± 1.0% cell death and after a 48-hour incubation was 17.0 ± 0.9% cell death. At the same concentration TRAIL induced 14.6 ± 0.7%–17.0 ± 0.8% apoptosis in a time-dependent manner in LNCaP cells. TRAIL concentrations higher than 100 ng/mL resulted in no significant increase in cytotoxic or apoptotic activity. These data confirmed that the LNCaP cell line is resistant to TRAIL-mediated apoptosis. Then the cytotoxic and apoptotic effects of EEP-P in combination with TRAIL were tested on LNCaP cells. After cotreatment of cancer cells with 25–50 *μ*g/mL EEP-P and 100 ng/mL TRAIL for 24–48 h the cytotoxicity ranged 20.4 ± 1.6%–66.9 ± 0.7% in a dose- and time-dependent manner. The cytotoxicity measured by MTT assay is shown in Figure 6(a). EEP-P cooperated with TRAIL to induce apoptosis in prostate cancer cells. When cells were treated with the same concentrations of EEP and TRAIL for 24–48 h, the percentage of apoptotic cells determined by annexin V-FITC staining using flow cytometry was elevated to 24.0 ± 0.8–69.8 ± 1.1% (Figure 6(b)). EEP-P sensitized the TRAIL-resistant LNCaP cells to TRAIL-mediated apoptosis. The annexin V-FITC staining, visualized by fluorescence microscopy, confirmed that EEP-P augments the apoptotic activity of TRAIL in LNCaP cells (Figure 6(c)). The necrotic cell death percentage of LNCaP cells incubated with EEP-P and/or TRAIL examined by Apoptest-FITC and LDH assay was near zero. 

### 3.4. EEP-P Upregulates Expression of TRAIL-R2 Receptor in LNCaP Cells

**Figure 7:**
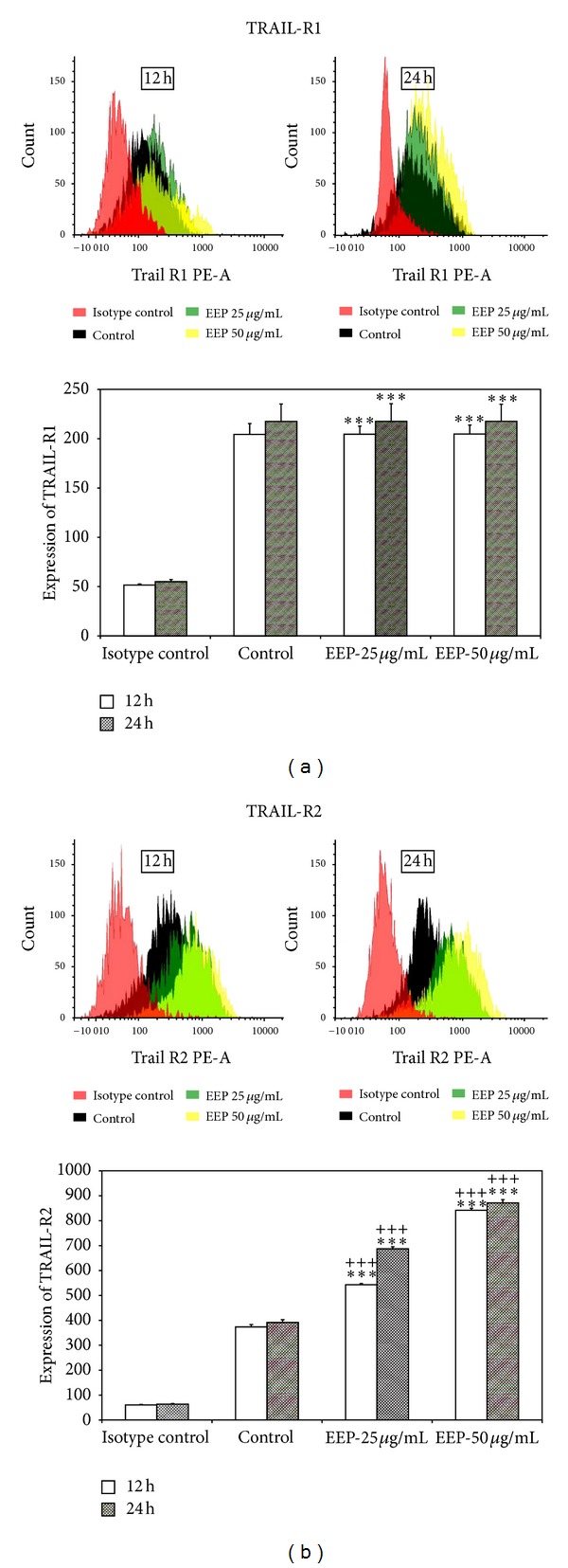
Effects of EEP-P on death receptor expression in LNCaP prostate cancer cells. Cells were incubated with 25–50 *μ*g/mL for 12–24 h. (a) TRAIL-R1 and (b) TRAIL-R2 expression on LNCaP cells treated with EEP-P measured by flow cytometry. The values represent mean ± SD of three independent experiments performed in quadruplicate (*n* = 12) shown as the average mean fluorescence (*** = *P* < 0.001 EEP-P compared to isotype control,  ^+++^ = *P* < 0.001 EEP-P compared to control) and histograms.

**Figure 8:**
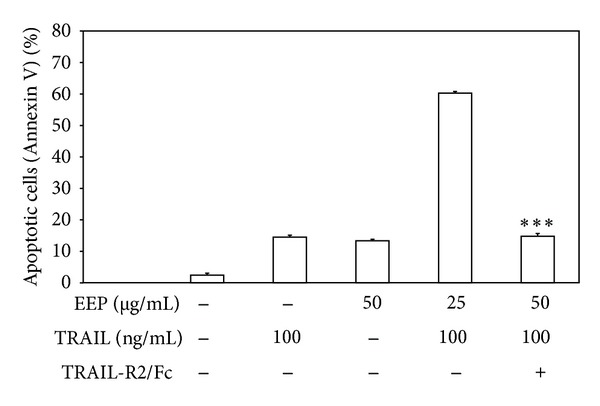
TRAIL-R2/Fc chimera block apoptosis induced by combination of EEP-P and TRAIL in LNCaP prostate cancer cells. Cells were incubated with 50 *μ*g/mL EEP-P and/or 100 ng/mL TRAIL with or without 1 *μ*g/mL TRAIL-R2/Fc chimera proteins for 24 h. Apoptotic cell death was detected by annexin V-FITC staining using flow cytometry. The values represent mean ± SD of three independent experiments performed in duplicate (*n* = 6) (*** = *P* < 0.001 compared to combination EEP-P+TRAIL).

The activation of death receptors on the cancer cell surface is critical for TRAIL-mediated apoptosis. Therefore, we analyzed the expression of death receptor TRAIL-R1 and TRAIL-R2 in LNCaP cells after a 12–24 h treatment with 25–50 *μ*g/mL EEP-P by flow cytometry (Figure 7). The high affinity death signaling TRAIL-R2 is more abundantly expressed in LNCaP cells than TRAIL-R1. Treatment with EEP-P significantly increased the expression of TRAIL-R2 but did not alter TRAIL-R1 expression on the cell surface. EEP-P induced TRAIL-R2 expression on LNCaP cells in a dose- and time-dependent manner. EEP-P sensitizes prostate cancer cells through TRAIL-R2 upregulation. To show that the induction of apoptosis caused by the cotreatment of EEP-P and TRAIL was mediated through TRAIL-R2, we used the TRAIL-R2/Fc chimera protein, which acts as a dominant negative against endogenous TRAIL-R2 receptor. The TRAIL-R2/Fc efficiently blocked apoptosis induced by cells EEP-P and TRAIL (Figure 8). These data indicate that the induction of TRAIL-R2 by EEP mediates the sensitization of LNCaP cells to TRAIL.

## 4. Discussion

Propolis extracts exert anticancer and chemopreventive properties by multiple mechanisms of action affecting apoptotic pathways in cancer cells. The role of propolis in host immune functions against tumor onset has become increasingly recognized in our understanding of the mechanisms of cancer prevention. EEP stimulates antitumor activity of TRAIL and enhances TRAIL-mediated immunity [9, 30]. In our opinion, the immunomodulatory effect of propolis could be evoked by the targeting of TRAIL-induced apoptosis in cancer cells. We showed that TRAIL-resistant prostate cancer cells can be sensitized by Polish or Brazilian EEP and its phenolic components [9, 30]. Because TRAIL-mediated apoptosis in LNCaP cells was augmented by EEP, we considered the possibility that propolis might influence the expression of DRs. There are two transmembrane agonistic receptors, TRAIL-R1 (DR4) and TRAIL-R2 (DR5), which bind ligand TRAIL by extracellular domains. DRs contain complete and functional intracellular death domains (DD) responsible for the activation of apoptotic pathway in cancer cells [21, 23]. Ligation of TRAIL to DRs activates the extrinsic apoptotic pathway, also known as the death receptor pathway [17]. Expression of TRAIL-R1 and/or TRAIL-R2 in cancer cells plays a critical role in intensity and/or duration of death receptor-mediated signaling in response to death ligand [19]. The decreased level of DRs in cancer cell surface causes TRAIL resistance [27]. Recent studies using affinity assays and phage displays of DR-selective TRAIL variants have revealed that TRAIL-R2 may have a more prominent role than TRAIL-R1 in TRAIL-mediated apoptosis [23, 28]. The flow cytometric analysis has shown significantly higher expression of TRAIL-R2 in LNCaP cells in comparison to TRAIL-R1. To explain the mechanism underlying the synergistic induction of apoptosis by propolis extract in LNCaP cells, we examined the effect of EEP-P on DRs expression. EEP-P markedly increased TRAIL-R2 protein level in LNCaP cells. Our previous findings demonstrated that the upregulation of TRAIL-R2 by Brazilian green propolis extract enhances TRAL-induced apoptosis in LNCaP cells [30].

Propolis significantly augmented the anticancer activity of TRAIL due to its phenolics [29]. It has been suggested that influence of TRAIL-R2 or TRAIL-R1 is a common response to treatment of cancer cells with compounds identified in propolis such as chrysin, apigenin, kaempferol, quercetin, or artepillin C. Chrysin and apigenin reverse TRAIL resistance in MDA-MB-231 breast cancer cells, HT-29 colon cancer cells, HepG2 hepatocellular cancer cells, SK-MEL-37 melanoma cells, and Capan-1 pancreatic cancer cells *via* increased expression of TRAIL-R2 and decreased expression of FLIP [43]. Apigenin augments TRAIL-induced apoptosis in Jurkat leukemia cells, DU145 prostate cancer cells, and DLD-1 colon cancer cells through upregulation of TRAIL-R2 and activation of Bid and caspase-8, -10, -9, -3 [44]. Quercetin strongly cooperates with TRAIL to trigger apoptosis in HepG2, SK-Hep, SNU-387, SNU-423, SNU-449, and SNU-475 hepatocellular cancer cells by increased expression of TRAIL-R2 and decreased expression of FLIP, in HT-29, SW-620, and Caco-2 colon cancer cells by upregulation of TRAIL-R1 and TRAIL-R2, induction of Bid and caspase-3 cleavage, and release of cytochrome *c* to the cytosol, in DU145 prostate cancer cells by upregulation of TRAIL-R2 and activation of caspase-9 and -3, in H460, H2009, H1299, and A549 lung cancer cells by increase of TRAIL-R2 expression, activation of caspase-8 and -3, and inactivation of Akt and survivin [45–48]. Induction of TRAIL-R1 and TRAIL-R2 expression and caspase-8, -10, -9, -3 activation in SW-480 colon cancer cells by kaempferol are sufficient to restore TRAIL sensitivity [49]. Artepillin C overcomes TRAIL-resistance in LNCaP prostate cancer cells by upregulation of TRAIL-R2, activation of caspase-8 and caspase-3, and the disruption of MMP [50].

Phenolic components contribute to overall cancer preventive and antitumor properties of propolis [9, 29, 30]. The tested sample of Polish propolis was rich in pinobanksin, chrysin, methoxyflavanone, *p-*coumaric acid, ferulic acid, caffeic acid, and their derivatives.

The TRAIL-induced apoptotic pathway in cancer cells may be a target for the chemopreventive activity of propolis and its phenolic ingredients. In this report, we demonstrated for the first time the mechanism by which EEP-P affects TRAIL-mediated apoptosis. EEP-P reverses TRAIL-resistance in LNCaP cells through upregulation of TRAIL-R2. These findings suggest that EEP-P supports TRAIL-mediated immunochemoprevention in prostate cancer cells.

## 5. Conclusion

Targeting TRAIL-induced apoptotic pathway in prostate cancer cells by EEP could be one of the mechanisms responsible for chemopreventive activity of propolis. Extract of Polish propolis sensitizes prostate cancer cells to TRAIL-mediated apoptosis through upregulation of TRAIL-R2 expression. 

## Figures and Tables

**Table 1:** The content (mg/g) and characterization of phenolic compounds of the ethanol extract of Polish propolis determined using their spectral characteristic in negative ions in LC-ESI/MS.

Peak	Retention time *t*_*r*_ (min)	(M−H)^−^	MS/MS fragments	Compound name*	Quantity (mg/g of propolis)**
1	2.26	179.0349	161.0241/135.0440	Caffeic acid^a^	3.90
2	2.53	121.0288	121.0288	Benzoic acid^bc^	0.86^d^
3	2.69	237.0772	163.0406/145.0284/117.0342	Ni	Tr
4	2.79	163.0406	119.0488	*p*-Coumaric acid^a^	28.65
5	2.88	267.0899	160.0154/133.0276	Ni	Tr
6	3.01	193.0492	178.0258/149.0613/134.0375	Ferulic acid^a^	22.01
7	3.18	357.0989	279.0655/147.0441	Ferulic acid derivative^c^	0.59^e^
8	3.31	295.0815	161.0241/133.0299	Ferulic acid derivative^c^	Tr
9	3.57	359.1129	163.0380/145.0284/119.0510	Coumaric acid derivative^c^	0.16^g^
10	3.72	389.1221	193.0492/134.0375/175.0399/160.0154	Ferulic acid derivative^c^	0.75^e^
11	3.88	279.086	145.0284/117.0342	Ni	Tr
12	4.15	415.1065	253.0730/179.0349/161.0241/135.0440	Caffeic acid derivative^c^	0.11^f^
13	4.27	355.1176	179.0349/163.0380/135.0440/119.0488	Caffeic and coumaric acids derivative^c^	Tr
14	4.61	399.1051	253.0697/179.0349/163.0380/119.0488	Coumaric and caffeic acids derivative^c^	1.83^g^
15	4.70	429.1214	253.0697/193.0492/161.0241/134.0375	Ferulic acid derivative^c^	2.12^e^
16	4.86	269.0469	151.0042/117.0342	Apigenin^a^	1.98
17	4.96	383.1137		Coumaric acid derivative^c^	3.97^g^
18	5.05	271.0616	253.0502/197.0596/161.0605/125.0241/107.0134	Pinobanksin^a^	12.78
19	5.10	383.1137	163.0406/119.0510	Coumaric acid derivative^c^	22.87^g^
20	5.20	413.1240	193.0492/163.0380/134.0375/119.0488	Coumaric and ferulic acids derivative (metoxy-)^c^	13.44^e^
21	5.27	443.1320	193.0492/163.0380/134.0375/119.0488	Coumaric and ferulic acids derivative (dimetoxy-)^c^	3.98^e^
22	5.44	259.1917	193.0492/163.0380/134.0375/119.0489	Ferulic acid derivative^c^	2.02^e^
23	5.63	283.0594	193.0492/134.0375	Ferulic acid derivative^c^	0.3^e^
24	5.73	441.1179	179.0349/163.0406/135.0440/119.0388	Caffeic and coumaric acids derivative^c^	Tr
25	5.79	315.0512	121.0288	Rhamnetin^a^	0.49
26	5.86	471.1305	297.1136/193.0492	Ni	Tr
27	6.25	425.1242	163.0406/119.0510	Coumaric acid derivative^c^	5.00
28	6.32	247.0982	179.0349/163.0380/135.0440	Coumaric and caffeic acids derivative^c^	0.31^f^
29	6.35	253.0502	143.0510	Chrysin^a^	6.56
30	6.41	269.0804	178.0258/163.0380/134.0375/119.0488	Ni	—
31	6.56	269.0435	171.0446/151.0042/117.0342	Galangin^a^	0.47
32	6.56	299.0572		Kaempferide^g^	Tr
33	6.66	313.0745	253.0502	Pinobanksin-3-O-acetate^b^	Tr
34	6.94	249.1131	161.0241/133.0299	Ni	—
35	7.13	253.0860	162.0308/151.0394/145.0284/117.0342	Methoxyflavanone^bc^	20.50^h^
36	7.25	283.0973	163.0380/145.0653/119.0488	Coumaric acid derivative^c^	10.49^g^
37	8.80	449.2537	361.2026/253.0502/121.0288	Benzoic acid derivative^c^	5.20^d^

Ni: not identified.

Tr: traces.

*a: confirmed by standard; b: confirmed by reference [41, 42]; c: confirmed by MS fragmentation.

**d: expressed as cinnamic acid; e: expressed as ferulic acid; f: expressed as caffeic acid; g: expressed as *p*-coumaric acid; h: expressed as apigenin.
